# Effects of CT-Xp Gene Knock down in Melanoma Cell Lines

**DOI:** 10.18632/oncotarget.921

**Published:** 2013-03-27

**Authors:** Otavia L. Caballero, Tzeela Cohen, Sita Gurung, Ramon Chua, Peishan Lee, Yao-Tseng Chen, Parmjit Jat, Andrew J. G. Simpson

**Affiliations:** ^1^ Ludwig Institute for Cancer Research, New York Branch at Memorial Sloan-Kettering Cancer Center, New York, USA; ^2^ Department of Pathology and Laboratory Medicine, Weill Cornell Medical College, New York City, New York, USA; ^3^ Department of Neurodegenerative Disease, UCL Institute of Neurology, Queen Square, London, UK; ^4^ Ludwig Institute for Cancer Research, 666 Third Avenue, New York, NY, USA

**Keywords:** Cancer/testis genes, GAGE, XAGE1, SSX, siRNA, melanoma

## Abstract

Cancer/testis (CT) genes are encoded by genes that are normally expressed only in the human germ line but which are activated in various malignancies. CT proteins are frequently immunogenic in cancer patients and their expression is highly restricted to tumors. They are thus important targets for anticancer immunotherapy. In several different tumor types, the expression of CT-X genes is associated with advanced disease and poor outcome, indicating that their expression might contribute to tumorigenesis. CT-X genes encoding members of the MAGE protein family on Xq28 have been shown to potentially influence the tumorigenic phenotype. We used small interfering RNA (siRNA) to investigate whether CT-X mapping to the short arm of the X-chromosome might also have tumorigenic properties and therefore be potentially targeted by functional inhibitors in a therapeutic setting. siRNAs specific to *GAGE*, *SSX* and *XAGE1* were used in cell proliferation, migration and cell survival assays using cell lines derived from melanoma, a tumor type known to present high frequencies of expression of CT antigens. We found that of these, those specific to *GAGE* and XAGE1 most significantly impeded melanoma cell migration and invasion and those specific to *SSX4* and *XAGE1* decreased the clonogenic survival of melanoma cells. Our results suggest that *GAGE*, *XAGE1* and *SSX4* might each have a role in tumor progression and are possible therapeutic targets for the treatment of melanoma and other malignancies.

## INTRODUCTION

Cancer/testis (CT) genes are normally expressed only in the human germ line and malignant cells [1]. Because of their restricted expression and immunogenicity, CT proteins are being used as targets in several therapeutic vaccination trials [2, 3]. The CT genes located on the X chromosome present the most tissue restricted expression [1] and most of them are encoded by multigene families that are organized in gene clusters. CT gene clusters are present on the telomeric end between Xq24 and Xq28, which includes CT1/*MAGEA*, CT6/*NY-ESO-1*, CT7*/MAGE-C1*, CT10*/MAGEC2*, and CT14/*SAGE*, and at a more centromeric position of X chromosome, Xp11.2-11.4, where CT4/*GAGE*, CT5/*SSX* and CT12/*XAGE1* genes are located [4].

There are relatively few clues regarding function of most of these proteins. Better insights in the function of these genes may uncover links between gametogenesis and tumor growth and could be indicative of their use in additional forms of anti-tumor therapies [1]. In several tumor types, the expression of CT-X genes is associated with advanced disease and poor outcome [5-16] and although these data indicate that CT gene expression might contribute to tumorigenesis, the biological role of these proteins in both germ line tissues and tumors remains poorly understood. Most functional investigations have focused on members of the MAGE proteins on Xq28. Several studies have shown that MAGE proteins are involved in cell survival, can increase tumorigenic properties of cells and may actively contribute to the development of malignancies [17-23]. However, the functional properties of CT-X genes mapping to the short arm of the X-chromosome (CT-Xp) remain poorly investigated. In this study, we used siRNA-mediated knock down in melanoma cell lines to evaluate the potential of CT genes on Xp as therapeutic targets.

## RESULTS

### Transfection of 27mers specific to CT-Xp antigens strongly and specifically suppressed gene expression in SK-MEL-37 cells.

**Figure 1:**
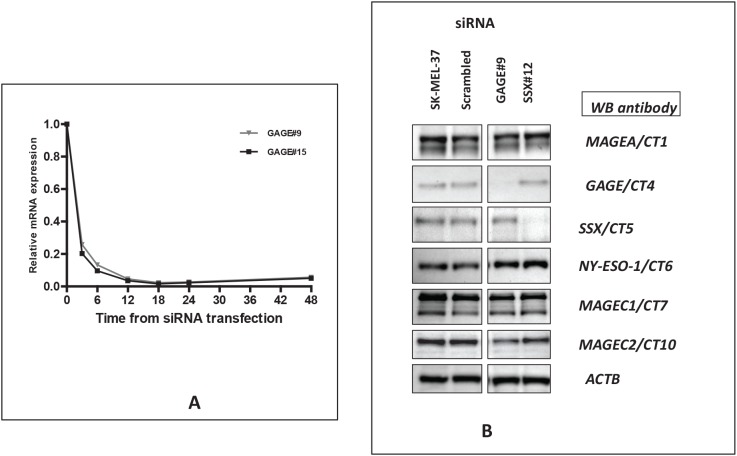
A – Kinetics of siRNA-mediated CT-X knockdown: SK-MEL-37 cells were transfected separately with10 nM of scrambled, GAGE#9 and #15 siRNAs and cells were harvested for real-time PCR 3, 6, 12, 18, 24 and 48 hours after transfection Relative quantification of gene expression (relative amount of target RNA) was determined using the equation 2^−ΔΔC^_T_ using the sample transfected with scrambled siRNA as calibrator. **B: Efficiency of siRNA-mediated CT-Xp knockdown: Western blot analysis was used to examine the effect of the specific siRNAs on CT-Xp expression at the protein level**. Reduction of protein levels to almost complete depletion was present 72 hours after transfection with all tested siRNAs.

We designed and tested siRNAs specific to the CT-Xp genes *GAGE/*CT4, *SSX/*CT5 and *XAGE/*CT12 (Table 1). The *GAGE* siRNAs were designed to target all members of the GAGE family; those specific to *XAGE1* target all isoforms of this gene, while both *SSX* siRNAs had 100% identity with *SSX4* only. These siRNA duplexes targeting the coding regions of the different CT-X and the siRNA specific to *HPRT1* were individually introduced into the SK-MEL-37 melanoma cell line and the effect on mRNA level examined by real-time quantitative RT-PCR analysis 24-48 hours post transfection. All siRNA duplexes examined produced a 91–99% reduction in CT-X mRNA compared with the control sample transfected with scrambled siRNA as a negative control (Table 2). In addition, we analyzed the effects of each siRNA duplex on the mRNA level of other CT-Xs, and little to no effect was observed compared with the scrambled control siRNA, suggesting that the effects of the 27mer siRNAs on these genes were sequence-specific. We also examined the kinetics of gene silencing and analyzed the levels of *GAGE* mRNA at 3, 6, 12, 18, 24 and 48 hours after transfection with *GAGE*-specific siRNAs (Figure 1A). Around 75-80% mRNA reduction could be observed as early as three hours after transfection at 10nM final duplex concentration.

**Table 1:** Characteristics of the 27mer siRNA duplexes designed for this study

Duplex name	Sense sequence (5'-3')	Antisense sequence (5'-3')	RefSeq	Sense position	Antisense position	Specificity2
GAGE#9	GUUCAGUGAUGAAGUGGAACCAGCA	UGCUGGUUCCACUUCAUCACUGAACUG	NM_001468	209	233	GAGE1,2,8,10,12
GAGE#15	GAACCAGCAACUCAACGUCAGGATC	GAUCCUGACGUUGAGUUGCUGGUUCCC	NM_001468	249	273	GAGE1,2,8,10,12
SSX#12	CAAGGUCACCCUCCCACCUUUCATG	CAUGAAAGGUGGGAGGGUGACCUUGAA	NM_005636	247	271	SSX4, SSX4B
SSX#19	CUUGUGUAUCCAUGCACCUACCUCA	UGAGGUAGGUGCAUGGAUACACAAGCC	NM_005636	892	916	SSX4, SSX4B, SSX6
XAGE1 #2	GACAGAAGAAGAUCAGGAUACAGCT	AGCUGUAUCCUGAUCUUCUUCUGUCUG	NM_133430	197	221	XAGE1
XAGE1 #9	AAGCUGAAACAACGCAAGCUGGUTT	AAACCAGCUUGCGUUGUUUCAGCUUGU	NM_133430	406	430	XAGE1

1 The sense strand has two terminal 3' nucleotides as DNA, and the remainder bases as RNA for preferential uptake of the antisense strand into RISC (RNA induced silencing) complex.

2 Only genes presenting 100% identity with the siRNA, as assessed with the BLAST tool at NCBI are listed.

**Table 2:** Degree and specificity of gene knock down

PCR Probe	MAGEA3	GAGE	SSX4	NY-ESO-1	MAGEC1	XAGE1
27mer duplex						
Scrambled	1	1	1	1	1	1
GAGE#15	0.823	0.056	1.112	0.556	0.814	0.821
GAGE#9	0.974	0.050	0.569	0.339	0.749	0.538
SSX#12	1.029	0.924	0.054	0.659	0.688	0.911
SSX#19	0.458	0.638	0.088	0.458	0.350	0.405
XAGE1#2	0.427	0.814	1.142	0.520	0.699	0.086
XAGE1#9	0.323	0.638	0.373	0.723	0.498	0.030

Western blot analysis was used to examine the effect of *GAGE* and *SSX*-specific siRNAs on CT-X expression at the protein level (Figure 1B). Reduction of protein levels to almost complete depletion was present 72 hours after transfection with all siRNAs tested. Similarly to the RT-PCR results, we found that the *GAGE* and *SSX* siRNAs do not alter the expression of the other CT-X proteins tested. No commercially available anti-XAGE1 antibody was found to be adequate for Western blotting analyses and our own attempts to produce anti-XAGE1 monoclonal or polyclonal antibodies failed. However, we assume that since in all other cases tested, the 27-mer induced gene knock down was very efficient at the protein level that it was for XAGE1 as well.

### Effects on of *GAGE*, *XAGE1* and *SSX4* knockdown on SK-MEL-37 proliferation and clonogenic survival.

**Figure 2:**
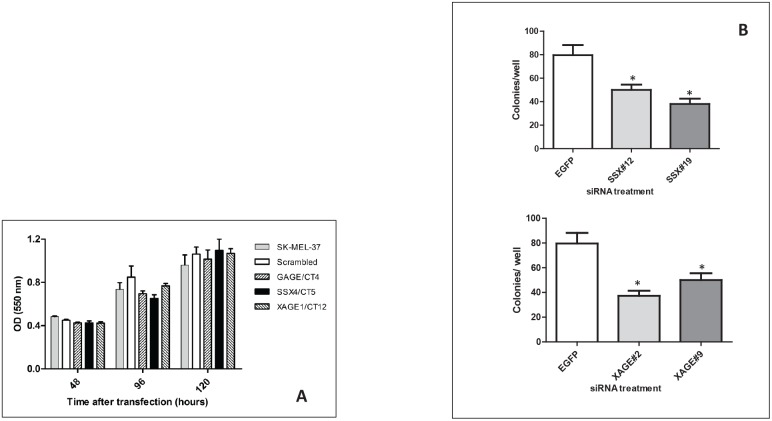
A: Effect of CT-X knockdown cell proliferation as determined by the MTT assay Results were representative of two experiments with each siRNA. The knock down levels in these experiments were confirmed by real-time PCR. Data are means±SD. **B: siRNA duplexes specific to** *SSX* **and** *XAGE1* **inhibit colony formation of SK-MEL-37 cell line**. Significantly reduced colony numbers transfected with SSX#12 and SSX#19 and XAGE1#2 and XAGE1#9 were observed as compared to cells transfected with non-targeting siRNA. All experiments were repeated at least three times and representative data are presented. The knock down levels of *SSX4* and *XAGE1* in these experiments were confirmed by real-time PCR. Bars, SD. *, *P* < 0.05 relative to non-targeting siRNA.

To investigate the biological result of depletion of CT-Xp by RNAi, we examined growth phenotypes of the melanoma cell line SK-MEL-37, which expresses high levels of the CT genes studied. First, the effect of CT-Xp knockdown on cell proliferation was determined by the MTT assay. The knockdown of the genes tested did not exert effects on cell proliferation, as determined by MTT assay performed with cells up to 120 hours after transfection (Figure 2A).

We next analyzed the ability of the siRNA-treated cells to form colonies between 10 and 14 days after transfection. The clonogenic assay relies on the ability of cells to form viable colonies derived from a single cell. In this colony formation assay, only 5-10% of control cells gave rise to colonies (plating efficiency). Depletion of *SSX4* and *XAGE1* significantly reduced the colony-forming ability of SK-MEL-37 cells to 50% or less of control levels (Figure 2B) (p<0.05). 27mer siRNA mediated silencing of *SSX4* and *XAGE1* using different duplexes (Table 1) that had similar specificity and efficiency of gene knock down (Table 2), equally reduced clonogenic survival and migration of SK-MEL-37 melanoma cell-line, reducing the possibility of off-target effects. Depletion of *GAGE* genes did not alter cell colony formation in SK-MEL-37.

### Effects on of CT-X knockdown on SK-MEL-37 migration and invasion.

**Figure 3:**
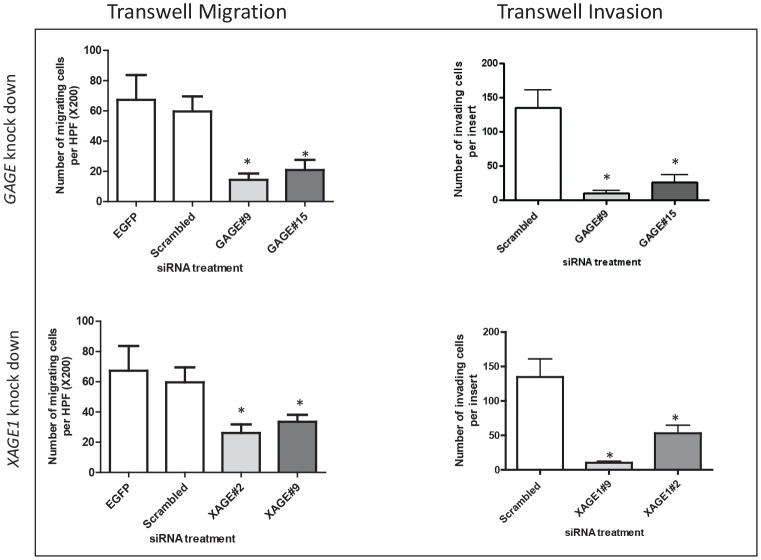
Depletion of *GAGE* and *XAGE1* in the melanoma cell line SK-MEL-37 results in reduced migration and invasion Transwell migration and invasion assays with SK-MEL-37 cells treated with nontargeting siRNA, GAGE-specific or XAGE1-specific siRNAs. All experiments were repeated at least three times and representative data are presented. The knock down levels of *GAGE* and *XAGE1* in these experiments were confirmed by real-time PCR. Bars, SD. *, *P* < 0.05 relative to non-targeting siRNA.

**Figure 4:**
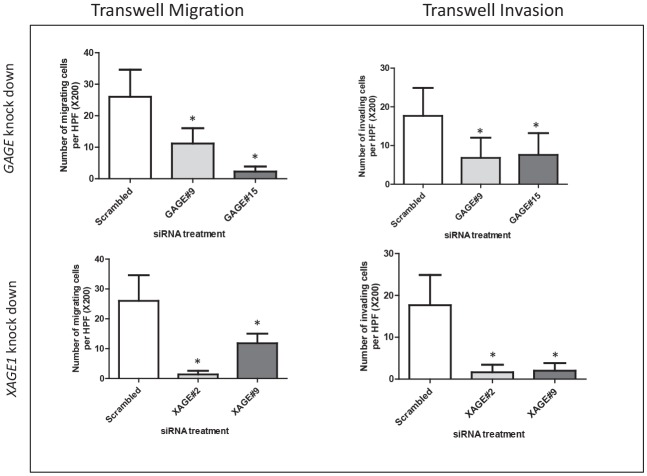
Depletion of *GAGE* and *XAGE1* in the melanoma cell line SK-MEL-119 results in reduced migration and invasion. Transwell migration and invasion assays with SK-MEL-119 cells treated with nontargeting siRNA, GAGE-specific or XAGE1-specific siRNAs. All experiments were repeated at least three times and representative data are presented. The knock down levels of *GAGE* and *XAGE1* in these experiments were confirmed by real-time PCR. Bars, SD. *, *P* < 0.05 relative to non-targeting siRNA.

To determine the possible role of CT-X in the migration of melanoma cells we used a transwell migration assay. siRNAs specific to *GAGE* and *XAGE1* significantly inhibited migration of melanoma cells (Figure 3A and B), while *SSX4* siRNA had no effect on cell migration. The influence of *GAGE* and *XAGE1* expression on cell invasion was also assessed using a modified Boyden chamber assay. A consistent and significant decrease in invasion of SK-MEL-37 with decreased *GAGE* and *XAGE1* levels was observed (Figure 3) (p<0.05). 27mer siRNA mediated silencing of *GAGE* and *XAGE1* using a different duplex equally reduced invasion of SK-MEL-37 (Figure 3). *GAGE* and *XAGE1* knockdown also significantly decreased transwell migration and invasion in another melanoma cell line (SK-MEL-119) (Figure 4), suggesting that these genes may have a positive effect on melanoma cell migration and invasion. However, the siRNAs specific to *XAGE1* had no effect on migration of a *XAGE1*-negative melanoma cell line, SK-MEL-124 (data not shown).

### *In vitro* effects of GAGE shRNA induction

**Figure 5:**
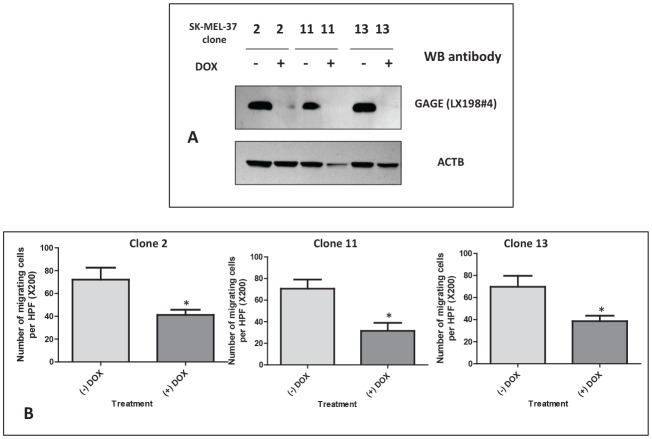
A: Western blot analysis of GAGE expression in double-stable Tet-On/GAGE shRNAmir clones, untreated (- DOX) or treated with doxycycline (+ DOX) Reduction of GAGE protein levels to almost complete depletion was present seven days after exposure to DOX. **B: Analysis of the migratory properties using a modified Boyden chamber assay of three double-stable Tet-On/GAGE shRNAmir clones, untreated (-DOX) or treated with doxycycline (+DOX)**. All experiments were repeated at least two times and representative data are presented. The knock down levels of GAGE in these experiments were confirmed by western blot. Bars, SD. *, *P* < 0.05 relative to non-DOX treated cell line.

To confirm the function of GAGE in the tumor migration and invasion processes, we used a melanoma cell system with inducible expression of GAGE shRNA (double-stable Tet-On/GAGE shRNAmir). Addition of doxycycline (DOX) induces GAGE shRNA in SK-MEL-37 and almost complete depletion of GAGE protein can be observed one week after exposure to DOX (Figure 5). Using this system, the role of GAGE in the migratory properties of tumor cells was examined in the transwell assay. We observed that cells exposed to DOX migrated significantly less than untreated cells (Figure 5B), while no effect was observed in the parental cells treated with DOX (data not shown). The inducible GAGE shRNA system was also tested in proliferation assays. Consistent with the results obtained with the transient siRNA transfections, no effect was observed on the rate of cell proliferation (data not shown).

### Effects on of *GAGE* and *XAGE1* knockdown on migration of cell lines from different origins.

To evaluate the effects of *GAGE* and *XAGE1* knockdown on the migration of additional *GAGE* and *XAGE1*-overexpressing cell lines (Table 3). Cell lines were tested for the expression of *GAGE* and *XAGE1* by RT-PCR, for their ability to migrate through transwell membranes and lastly for the efficiency of the transfection using the GAGE and XAGE1 siRNAs and Lipofectamine 2000. For *XAGE1*, in addition to the two melanoma cell lines shown previously (Figures 3 and 4), transwell migration was also significantly reduced in two other melanoma cell lines, two lung cancer cell lines and two prostate cancer cell lines. Neither of the two *XAGE1*-specific siRNAs had effect on migration of SK-MEL-124, which does not express *XAGE1*. While treatment with XAGE1 siRNA decreased migration in all *XAGE1* expressing cell lines tested, the treatment with GAGE-specific siRNA failed to decrease migration in three cell lines tested (LM-MEL-34, A172 and 22RV1) that expressed high levels of GAGE, although gene knock down, assessed by real-time PCR, was achieved.

**Table 3:** Effect of GAGE and XAGE1 knock down on migration of cell lines from different origins

Cell line	Origin	GAGE siRNA (effect on migration) 1	GAGE status^2^	XAGE1 siRNA1 (effect on migration)	XAGE1 status
SK-MEL-37	Melanoma	Decreased	+++	Decreased	+++
SK-MEL-119	Melanoma	Decreased	+++	Decreased	+++
LM-MEL-34	Melanoma	No effect	+++	NT	+++
SK-MEL-128	Melanoma	NT	+++	Decreased	+++
SK-MEL-124	Melanoma	NT	++	No effect	Negative
SK-MEL-131	Melanoma	NT	+++	Decreased	+++
U343MG	Glioma	Decreased	++	NT	Negative
A172	Glioma	No effect	+++	NT	+
SK-LC-19	Lung	Decreased	+++	Decreased	+++
SK-LC-5	Lung	NT	+	Decreased	+++
DU145	Prostate	No effect	Negative	Decreased	+++
22RV1	Prostate	No effect	++	Decreased	+++

NT: not tested

1 As determined by transwell assays

2 GAGE and XAGE1 status were determined by semiquantitative RT-PCR and evaluation of the intensity of the band in ethidium bromide stained agarose gels.

## DISCUSSION

Overall, our results suggest that inhibition on *SSX4*, *XAGE1* and *GAGE* expression in cancer cell lines interferes with tumor cell migration and/or reduce cell viability. We demonstrate that the observed RNAi-induced phenotype is probably a result of the suppression of CT-antigen expression and not an off-target effect. The finding that multiple siRNAs targeting different regions of the same gene have the same phenotypic effect indicates that these effects are indeed dependent on gene depletion.

Effects of members of the MAGEA family on survival of cancer cell lines have been shown before, using a similar approach used in our study [20-22, 24, 25], but the effects of depletion of GAGE, XAGE1 and SSX4 have not been previously investigated.

SSX proteins are thought to act as transcriptional corepressors. They were identified as fusion partners of the *SS18* gene in synovial sarcomas carrying t(X;18) translocations [26], which typically, result in fusion of the 78 most C-terminal amino acids of SSX genes to SS18, replacing its eight most C-terminal amino acids. SSX presents two transcriptional repressor domains, a Kruppel-associated box (KRAB) and an SSX repressor domain (SSXRD), both retained in the SS18-SSX fusion proteins [27]. Both *SS18* and *SSX* gene products, together with the fusion proteins, are localized in the nucleus but lack obvious DNA binding motifs. SS18-SSX was shown to block the tumor-suppressive function of p53 in the absence of inactivating p53 mutations by increasing its degradation, therefore promoting cell survival [28]. We speculate that the effects of *SSX4* inhibition seen in this study may be analogous to the effects of inhibition of the SS18-SSX fusion proteins.

The XAGE family of genes was first identified in a search for expressed sequence tags (ESTs) homologous to another CT gene, CT16/*PAGE-4*. This approach led to the identification of three novel *PAGE-GAGE*-related genes termed *XAGE-1*, *-2* and *-3* [29]. The *GAGE*s, *PAGE*s, and *XAGE*s form one large supercluster of related genes, which are expressed in various reproductive tissues as well as in different tumors [29]. One transcript variant (XAGE-1b), was identified as a dominant antigen recognized by sera from lung adenocarcinoma patients [30]. XAGE-1b mRNA expression was also observed in 26% of prostate cancer specimens [31].

*GAGE-1* was the first gene of the GAGE family to be identified from the melanoma cell line MZ2-Mel. Subsequent screening of the MZ2-MEL cDNA library with a *GAGE-1* probe identified five cDNAs, designated *GAGE-2*, -*3*, -*4*, -*5*, and -*6* sharing nucleotide identities with the *GAGE-1* sequence. *GAGE-1* differs from these other *GAGE* genes by the presence of a 143-bp insertion. The *GAGE* genes are located on chromosome Xp11 Protein products of GAGE genes have more than 95% of homology. GAGE gene transcripts have been found in numerous types of cancers, most frequently in melanomas and lung adenocarcinomas [32, 33], in which up to 54% of specimens were found to express GAGE, as well as in gastric cancers [34] and hepatocellular carcinomas [35]. Using a panel of mAbs, GAGE protein expression was identified in specimens of malignant melanoma, breast carcinoma, bladder carcinoma, lung carcinoma, liver carcinoma, thyroid carcinoma, mesothelioma and germinal cell cancers [36]. GAGE expression in melanoma cell lines ranged from 41% to 58% and in melanoma tissues from 22% to 53%. Immunohistochemical analysis of melanoma tumors revealed a rather heterogeneous expression of GAGE resulting in individual positive cells or foci of stained cells. Furthermore, autoantibodies against GAGE family proteins were detectable in 6% of melanoma patients [33]. GAGE has been correlated with poor prognosis in stomach cancer, esophageal carcinoma and neuroblastoma [34, 37, 38]. The function of GAGE proteins remains largely unknown, although antiapoptotic properties of GAGE-7 have been reported [39, 40].

We observed inhibition of cell migration and invasion in this study following the knock down of *XAGE1* and *GAGE* genes, both members of the GAGE family indicating that targeting of these genes may be useful for cancer treatment. The effects of *XAGE1* inhibition were very consistent, not only in melanoma cell lines, but also in cell lines from other tumors, including lung adenocarcinomas and prostate cancer that were shown to present frequent expression of XAGE1 [30, 31]. Although the mechanism of the impairment of migration following *XAGE1* or *GAGE* knock down was not yet elucidated, these results warrant further investigation of CT-X as therapeutic targets for melanoma and other malignancies.

## MATERIAL AND METHODS

### Cell lines and tumor tissues

The cell lines SK-MEL-37, SK-MEL-119, SK-MEL-124, SK-MEL-128 and SK-MEL-131 were obtained from the cell culture bank of the New York Branch of the Ludwig Institute for Cancer Research. They were maintained in RPMI medium containing 10% fetal bovine serum (FBS) and non-essential amino acids. These cell lines were selected for study because they express high levels of the CT genes analyzed.

### 27mer siRNA oligonucleotide design – Dicer substrate RNAs

Dicer-Substrate RNAs are chemically synthesized 27-mer RNA duplexes that are optimized for Dicer processing and show increased potency when compared with 21-mer duplexes [41, 42]. The duplexes were chosen by a rational design algorithm that integrates both traditional 21-mer siRNA design rules as well as new 27-mer design criteria available at http://www.idtdna.com/Scitools/Applications/RNAi/RNAi.aspx. The approximately 20 options identified by the algorithm in each case were optimized at several levels. We first level aimed to exclude off-target complementarity. This was undertaken with the BLAST tool at NCBI adjusted for analyzing short sequences (http://www.ncbi.nlm.nih.gov/BLAST/). Sequences were excluded if total or partial complementarity with other genes was noted. Further selection was based on published criteria for selection of active siRNA [43, 44]. siRNA sequences were designed to target all isoforms and possible members of the gene families. The selected siRNA sequences are shown in Table 1.

### Gene downregulation by 27-mer siRNAs

siRNAs were purchased from IDT (Integrated DNA Technologies, Coralville, IA). The RNAs were resuspended in the RNase-free Duplex Buffer (IDT, Coralville, IA) to 20 μM final concentration; vortexed thoroughly, microfuged and heated to 94°C for 2 minutes, and allowed to cool to room temperature to ensure that the formation of duplexes. Once hydrated, duplexes were stored at -80°C in aliquots. A scrambled universal negative control RNA duplex (DS Scrambled Neg) and a siRNA specific to EGFP, both absent in human, mouse, and rat genomes, and a positive control Dicer-Substrate RNA duplex (HPRT-S1 DS Positive Control), targeting *HPRT* (hypoxanthine guanine phosphoribosyltransferase 1) and prevalidated to give >90% knockdown of *HPRT* when transfected at 10 nM concentration, were also purchased from IDT (IDT, Coralville, IA) and used as negative and positive controls, respectively. siRNA duplexes were used to transfect the melanoma cell lines cells using Lipofectamine™ 2000 (Invitrogen, Carlsbad, CA) following the manufacturer's recommended protocols to a final concentration of 10nM. Briefly, 1 × 10^5^ cells were seeded into 60mm dishes containing antibiotic-free medium and incubated overnight to reach a density of 50-70%. For each dish, 5 μL of 10μM siRNA solution was mixed with 500 μl of OPTI-MEM I. The mixture was then combined with a solution of 10 μL lipofectamine 2000 in 500 μL OPTI-MEM I and, after 20 minutes at RT, the mixture was applied to the cells, and the final concentration in the dish was 10 nmol/l for each siRNA. After incubation for 24 h at 37°, the medium was changed to RPMI-1640 supplemented with serum and cells were then cultured for an additional 24-48 h at 37° before analysis.

### RNA extraction and reverse transcription

Total RNA from the cell pellets was isolated using the RNeasy Mini Kit (Qiagen, Valencia, CA). RNA amounts were estimated by spectrophotometric analysis (Nanophotometer, Implen, Germany). One μg of RNA was reverse transcribed into cDNA by using an Omniscript RT kit according to the manufacturer's protocol using oligo (dT)_18_ primers.

### Semi-quantitative reverse transcription -PCR

RT-PCR was undertaken with Jump-Start master mix (Sigma) plus 10 pmol of each of the following primers (5'-3'): GAGE F: GACCAAGACGCTACGTAG, GAGE R: CCATCAGGACCATCTTCA, XAGE1F: TCCCAGGAGCCCAGTAATGGAGA, XAGE1R: CAGCTTGTCTTCATTTAAACTTGTGGTTGC, ACTBF: AAATCTGGCACCACACCTTC, ACTBR: CACTGTGTTGCCGTACAGGT. The amplification involved three stages in which the annealing temperature was higher (60°C) in the first ten cycles and reduced in two degrees in the following stage (ten cycles) and other two degrees in the last 15 cycles and involved an initial denaturation at 94°C for 5min. Each cycle consisted of a denaturation step at 94°C for 30s, followed by 30 s at the annealing temperature and extension at 72°C for 30 s followed by a final 7-min extension. Controls without DNA and using cDNA of testis as a positive control were carried out for each set of reaction. PCR products were loaded onto 2% agarose gels, stained with ethidium bromide and visualized by UV illumination. The predicted sizes of the PCR products were 243 bp and 257 bp for GAGE and XAGE1, respectively. A 644 fragment from *ACTB* was amplified as an endogenous control.

### Quantitative real-time reverse transcription-PCR

cDNA samples were run in duplicate for the genes of interest and for the reference gene within the same experiment using the Applied Biosystem apparatus 7500 Fast Real-Time PCR system and Taqman platform (Applied Biosystems, Foster City, CA). *TFRC* was amplified as an internal reference gene. The PCR primers and probes for all tested genes (MAGEA3, Hs00366532_m1; GAGE1, Hs00275620_m1; SSX4, Hs00171942_m1; NY-ESO-1, Hs00265824_m1; MAGEC1, Hs00193821_m1; XAGE1, Hs00220764_m1) and *TFRC* internal control gene (4326323E) were purchased from Applied Biosystems. Primers used for PCR amplification were chosen to encompass intron between exon sequences to avoid amplification of genomic DNA (Applied Biosystems, Foster City, CA). The gene-specific probes were labeled with the reporter dye 6-FAM at the 5'-end. The *TFRC* probe was labeled with a reporter dye (VIC) to the 5'-end of the probe and all probes had minor groove binder/nonfluorescent quencher at the 3'-end of the probe (Applied Biosystems, Foster City, CA). The PCR conditions were 95°C for 10 minutes followed by 40 cycles at 95°C for 15 seconds and 60°C for 1 minute. Duplicate threshold cycles (CT) were averaged for each sample. Relative quantification of gene expression (relative amount of target RNA) was determined using the equation 2^−ΔΔCT^.

### Determination of rate of cell proliferation

The rate of proliferation was determined using the 3-(4,5-dimethyl thizol-2-yl) 2,5-diphenyl tetrazolium bromide (MTT) assay (Roche, Indianapolis, IN). Cells (5×10^3^cells per well) were incubated in 96-well plates and maintained in complete medium 24 h after transfection. After 48, 96 and 120 hours, 10 μL of sterile MTT dye was added to the cells and incubated for 4 hours at 37°C, and then 100 μL of solubilization buffer was added. Spectrometric absorbance at a wavelength of 550 nm was measured on an enzyme immunoassay analyzer (Molecular Devices) after overnight incubation. Experiments were performed at least three times, with six replicate measurements, and data are presented as the average OD ± SD.

### Migration and invasion assays

Cell migration and invasion were assessed in 12-well Boyden Chambers (BD Biosciences, San Diego, CA) according to the protocol of the manufacturer. Invasion assays were carried out in chamber equipped with an 8 μm polycarbonate membrane coated with Matrigel. Briefly, cells were serum-starved for 2 hr, and 500 μl containing 25,000 cells in medium supplemented with 1% FBS were loaded into the upper chamber. The lower chamber contained medium supplemented with 10% FBS as the chemoattractant. Cells were incubated at 37°C overnight, fixed in 10% formalin for 20 min and stained with 0.2% crystal violet (Fisher Scientific, Pittsburgh, PA). Non-invading cells on the top of the membrane were wiped off using cotton swabs, and invading cells affixed to the underside of the membranes on each insert were counted at 100 × magnification in 10 random areas. The migration assay was done in a similar fashion except the 8.0-μm pore size membrane inserts were not coated with Matrigel. Results were expressed as mean ± SD. Experiments were performed at least three times.

### Colony formation assay

At 48h after transfection with each siRNA, cells were trypsinized, counted and 1,000 cells were seeded in duplicate in 6-well plates and allowed to form colonies for 2 weeks. The colonies were fixed with 10% formalin and stained with 0.2% crystal violet (Fisher Scientific, Pittsburgh, PA). The number of colonies with 30 cells or larger than 1mm in diameter in each well was counted. Experiments were repeated at least three times.

### Western blotting analyses

Cells were harvested and washed with cold phosphate-buffered saline solution, and total proteins were extracted in the extraction buffer (50 mM Tris-Cl pH 7.4, 0.15 M NaCl, 2mM EDTA 1% NP40), containing protease inhibitors (Protease Inhibitor Cocktail, Roche, Indianapolis, IN). Equal amounts of protein (20 μg per lane) were mixed with an equal volume of 2x loading buffer (125 mM Tris-HCl pH 6.8, 4% SDS, 10% glycerol, 0.006% bromophenol blue, 2% β-mercaptoethanol), incubated at 95°C for 3 min, and loaded in 10% SDS Bis-Tris gels (Invitrogen, Carlsbad, CA). After electrophoresis, proteins were transferred to nitrocellulose membranes. The membranes were blocked by incubation in PBST (PBS 0.1% Tween 20) 3% bovine serum albumin (BSA) for 1 h, then incubated with the primary antibody overnight at 4°C in PBST 1% BSA. After washing four times in PBST, the membranes were incubated either with peroxidase-conjugated anti-rabbit or anti-mouse IgG (Jackson Immunoresearch, Bar Harbor, ME) for 1 h at room temperature. Antibody binding was detected using the system Western Lightening Chemiluminescence Reagent Plus (Perkin Elmer, Emeryville, CA). The antibodies used were: monoclonal antibodies anti-NY-ESO-1 (E978, Sigma-Aldrich, St. Louis, MO), anti-MAGEC1 (CT7.33, Sigma-Aldrich, St. Louis, MO), anti-MAGEC2 (CT10#5), anti-GAGE LX198, anti-SSX (LX#1), anti-MAGEA (6C1, Santa Cruz Biotechnology, Santa Cruz, CA) and a rabbit polyclonal anti-actin (20-33, Sigma-Aldrich, St. Louis, MO).

### Establishment of the Double-stable Tet-On/GAGE shRNAmir cell lines

The double-stable Tet-On/GAGE shRNAmir SK-MEL-37 cell line was established using the Tet-On Advanced Inducible Gene Expression System (Clontech Laboratories). SK-MEL-37 was initially transfected with 5 μg of the tetracycline-controlled transactivator (pTet-On advanced) encoding the G418-resistant gene. After selection, a clone that presented high levels of expression of the transativator as assessed by the transfection of a reporter plasmid containing tetracycline response elements (TRE) within the promoter was identified. The pTet-On advanced cells were then submitted to retroviral infection with the microRNA-adapted retroviral vector pTMP (Open Biosystems), containing TET responsive promoter and the puromycin-resistant gene. Double-stable cells were then selected and further screened for GAGE protein expression by Western blot using the anti-GAGE LX198 antibody four days after exposure to 1μg/ml of doxycycline. For cloning in pTMP, a standard GAGE 21mer was created from the dicer substrate RNAi (GAACCAGCAACUCAACGUCAGGATC) by removing bases on the 3' end of the sense strand and on the 5'end of the antisense strand.

### Statistical analyses

Statistical comparisons were performed using analysis of variance for analysis of significance between different values using GraphPad Prism software (San Diego, CA). Values are expressed as mean with SD from an experiment representative of at least three separate experiments, and differences were considered significant at a *p* value of less than 0.05.
